# Circulating Tumor DNA Analysis: Clinical Implications for Colorectal Cancer Patients. A Systematic Review

**DOI:** 10.1093/jncics/pkz042

**Published:** 2019-06-19

**Authors:** Sander Bach, Nina R Sluiter, Jamie J Beagan, Joost M Mekke, Johannes C F Ket, Nicole C T van Grieken, Renske D M Steenbergen, Bauke Ylstra, Geert Kazemier, Jurriaan B Tuynman

**Affiliations:** 1 Department of Surgery, Cancer Center Amsterdam, Amsterdam UMC, Vrije Universiteit Amsterdam, Vrije Universiteit, Amsterdam, the Netherlands; 2 Department of Pathology, Cancer Center Amsterdam, Amsterdam UMC, Vrije Universiteit Amsterdam, Vrije Universiteit, Amsterdam, the Netherlands; 3 Medical Information Specialist/Literature Researcher Medical Library, Amsterdam UMC, Vrije Universiteit Amsterdam, Vrije Universiteit, Amsterdam, the Netherlands

## Abstract

**Background:**

Liquid biopsies could improve diagnosis, prognostication, and monitoring of colorectal cancer (CRC). Mutation, chromosomal copy number alteration, and methylation analysis in circulating tumor DNA (ctDNA) from plasma or serum has gained great interest. However, the literature is inconsistent on preferred candidate markers, hampering a clear direction for further studies and clinical translation. This review assessed the potential of ctDNA analysis for clinical utility.

**Methods:**

A systematic review according to the Preferred Reporting Items for Systematic Reviews and Meta-analyses guidelines was conducted up to December 3, 2018, followed by methodological quality assessment. Primary endpoints were accuracy for detection, prognostication, and monitoring.

**Results:**

Eighty-four studies were included. For CRC detection, sensitivity was 75% using ctDNA mutation analysis and up to 96% using copy number analysis. Septin 9 (*SEPT9*) hypermethylation analysis showed sensitivities of 100% and specificities of 97%. Regarding prognostication, ctDNA *KRAS* mutations were associated with oncological outcome and could predict response to anti–epidermal growth factor receptor therapy. For monitoring, sequential ctDNA *KRAS* mutation analysis showed promise for detection of relapses or therapy resistance.

**Conclusions:**

This comprehensive overview of ctDNA candidate markers demonstrates *SEPT9* methylation analysis to be promising for CRC detection, and *KRAS* mutation analysis could assist in prognostication and monitoring. Prospective evaluation of marker panels in clinical decision making should bring ctDNA analysis into practice.

Colorectal cancer (CRC) is the third most common cancer in the Western world (1,2) and the incidence is still rising (3). In recent decades, oncological outcomes have improved because of the implementation of screening programs, improvement of surgical procedures, and introduction of novel systemic regimens. However, CRC is still the second leading cause of cancer-related death (1,2). Further innovation is needed to improve diagnosis, patient-specific treatment selection, and disease monitoring.

The stage of disease at diagnosis is the most important prognostic factor for survival in CRC (4). It is therefore of utmost importance to detect CRC at an early stage, which requires improved screening approaches. The value of current screening methods is hampered by the low sensitivity of the fecal occult blood test (FOBT) and the invasive nature and costs of colonoscopy (5).

A second challenge concerns selection of the most suitable treatment, warranting better prognostic markers. The current decision process for systemic therapy is largely based on clinicopathological characteristics, leaving a substantial number of patients under- or overtreated. Genetic subtyping (6) and expression profiling (7) enhance patient selection. However, improved approaches are needed to further subclassify patients by their risk of recurrence and suitability for adjuvant therapies.

A third major area of interest is disease monitoring after initial curative treatment or during systemic therapy. Up to 40% of CRC patients will experience disease recurrence despite curatively intended treatment (8). Unfortunately, recurrences are often detected at advanced stages, excluding these patients from potentially curative rescue treatments. Current follow-up consists of serial carcinoembryonic antigen (CEA) measurements in serum, imaging, and colonoscopy (9). Unfortunately, the value of CEA for follow-up is limited by its low accuracy (10,11), with only marginal benefit observed when combined with computed tomography (CT) scans (12). The value of CT imaging is limited to the detection of large lesions, illustrated by a sensitivity of 11% for nodules smaller than 5 mm (13). Colonoscopy provides a high level of sensitivity (>95%) but can evaluate only endo luminal disease (5). These issues stress the urgent clinical need for a robust and noninvasive diagnostic marker facilitating CRC detection and prediction of treatment response.

Liquid biopsies are a rapidly developing field of research focused toward the analysis of cancer biomarkers isolated from nonsolid tissues. Various tumor-derived products can be detected in blood, including circulating tumor cells, circulating tumor DNA (ctDNA), circulating RNAs, exosomes, and tumor educated platelets (14–16). Of these tumor-derived products, ctDNA has been investigated most extensively and has shown promising accuracies for cancer detection (17–20). These DNA fragments originate from tumor cells and are released into the circulation through apoptosis, necrosis, and secretion (17). Accordingly, tumor-specific (epi-)genetic alterations such as driver mutations, chromosomal copy number alterations (CNAs), and methylation can be detected in ctDNA and could be of high value for cancer detection, prognostication, and treatment monitoring (17–20).

The primary challenge of ctDNA analysis is to detect tumor-derived molecules in a high background of cell-free DNA (cfDNA) from healthy cells. Currently, ctDNA detection techniques mainly revolve around real-time polymerase chain reaction (PCR) and sequencing approaches (14,15). Allele-specific quantitative PCR has a high sensitivity for ctDNA detection, with a detection limit of 0.014–0.004% (21). Emulsion PCR methods such as droplet digital PCR (ddPCR) and beads, emulsion, amplification, and magnetics are most sensitive, with a detection limit of 0.01–0.001% (22,23). The disadvantage of PCR-based methods is the limited number of foci that can be assessed, relying on the initial identification of patient-specific solid-tumor tissue alterations. Sequencing platforms including next-generation sequencing (NGS) allow for broader genomic coverage. However, this method is time consuming and expensive, hampering clinical implementation. An overview of the main methods to detect ctDNA is depicted in Figure 1.

**Figure 1.**
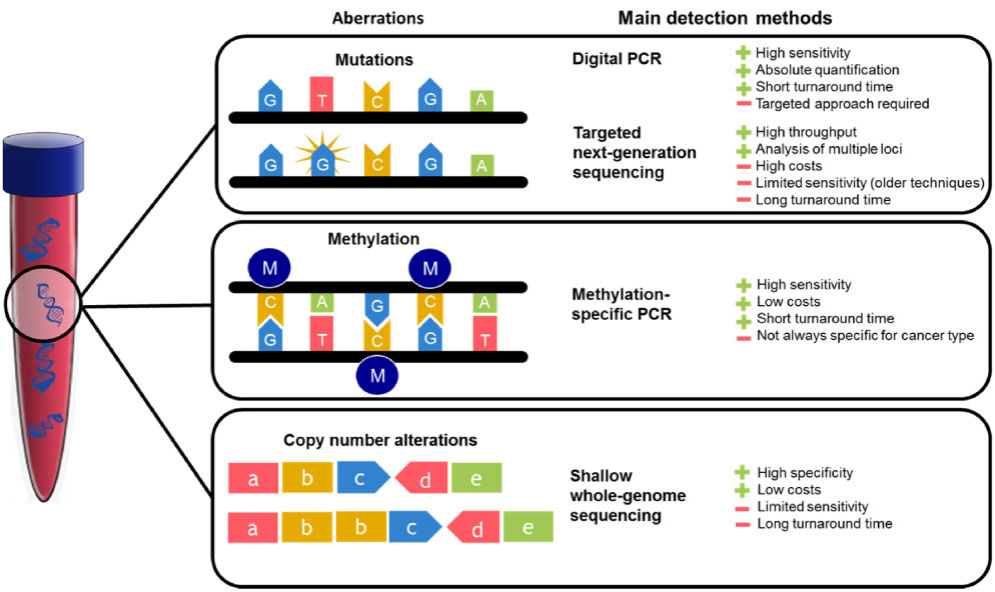
The three types of circulating tumor DNA aberrations covered in this review. For every DNA aberration, commonly used techniques to determine its presence in plasma or serum are depicted. PCR = polymerase chain reaction.

Several Food and Drug Administration (FDA)–approved assays are commercially available for ctDNA-based cancer diagnostics, including a PCR kit for detection of epidermal growth factor receptor (EGFR) mutations in non-small cell lung cancer patients (Cobas v2) (24) and a PCR assay measuring methylated *SEPT9* in blood to detect CRC (Epi ProColon) (25). Copy number analysis of circulating DNA is currently routine diagnostic practice in several countries, including the Netherlands, for noninvasive prenatal testing (26). Numerous studies claim a potential clinical role for ctDNA, but the diverse and sometimes contradictory results and recommendations hamper widespread translation into daily practice of CRC patients. Therefore, the aim of this study is to systematically review the current literature on the potential role of ctDNA mutation, copy number, and methylation analysis for CRC diagnosis, prognostication, and monitoring.

## Materials and Methods

### Search Strategy

A systematic literature review was conducted according to the Preferred Reporting Items for Systematic Reviews and Meta-analyses statement (27). Systematic searches were performed in the bibliographic databases PubMed, Embase.com, and Clarivate Analytics/Web of Science up to December 3, 2018, by SB, NRS, and JCFK (Supplementary Table 1). The search query included indexed terms and free-text words for “DNA” and “variation” or “methylation” and “blood” or “serum” and “colorectal cancer.”

### Study Selection

Screening and study selection was independently performed by three reviewers (JMM, NRS, SB). If necessary, articles were discussed to achieve consensus. All full-text articles in English, Dutch, French, German, or Russian on ctDNA mutation, copy number, or methylation analysis in the serum or plasma of CRC patients were considered eligible. Human studies assessing therapy-naive patients with a minimum age of 18 years that allowed determination of sensitivity were included. Literature reviews, case reports, and studies in which ctDNA analysis was performed in fewer than 10 CRC patients or in patients with hereditary CRC or inflammatory bowel disease were excluded. If overlapping data were reported, either the most recent study or that with the most complete data on our outcomes of interest was included.

### Data Extraction

Primary outcomes were sensitivity and specificity of ctDNA analysis for CRC detection, subdivided according to several clinical settings: diagnosis, prognostication, and monitoring. Sensitivity was defined as the percentage of CRC patients in whom a specific ctDNA aberration was detected. Specificity was defined as the percentage of healthy control individuals without detected ctDNA. Additionally, the technical concordance was extracted, defined as the percentage of agreement between ctDNA and solid-tumor tissue analysis. Data on single mutations in sequencing panels were extracted if two or more studies reported this mutation.

### Quality Assessment

Risk of bias assessment of all included studies was independently performed by three reviewers (JMM, NRS, SB). Risk of bias was scored as low, high, or unclear using the validated Quality Assessment of Diagnostic Accuracy Studies 2 tool (QUADAS-2) (28). Custom criteria were created, and agreement among reviewers was initially determined in a pilot of 10 studies. Disagreement was resolved by discussion with all reviewers present (JMM, NRS, SB). To ensure high-quality assessment of the described literature, articles were excluded from further analysis in case one domain was scored as “high” in combination with “unclear” or “high” risk at a second domain of the QUADAS-2. Review Manager 5 software (The Nordic Cochrane Centre, Copenhagen, Denmark) was used for managing the QUADAS-2 results.

## Results

**Figure 2.**
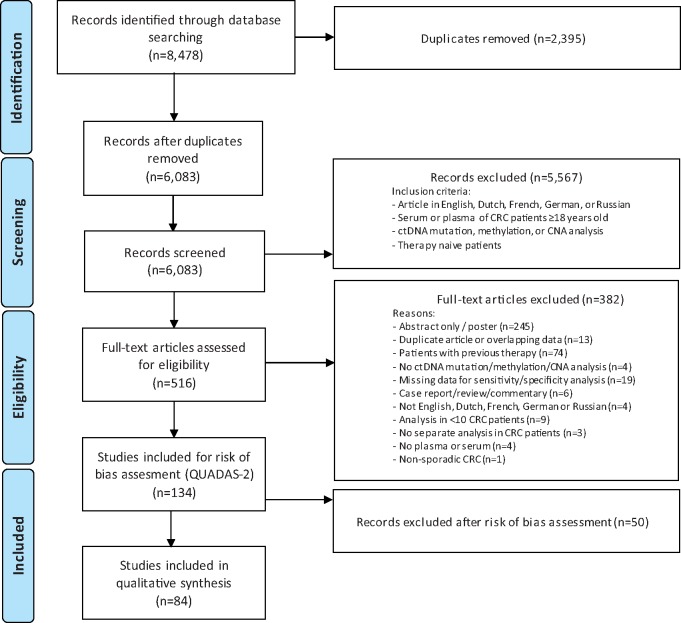
The Preferred Reporting Items for Systematic Reviews and Meta-analyses flowchart for inclusion of the studies. The risk of bias assessment using the Quality Assessment of Diagnostic Accuracy Studies 2 (QUADAS-2) was incorporated in the flowchart. CNA = copy number alteration; CRC = colorectal cancer; ctDNA = circulating tumor DNA.

The search identified 8478 eligible abstracts. After removal of duplicates, 5567 studies were excluded by title and abstract screening. Subsequently, 382 articles were excluded by full-text evaluation, leaving 134 studies, all in English, for risk of bias assessment. Figure 2 depicts the study selection procedure.

Fifty studies were excluded based on quality assessment using QUADAS-2, leading to the inclusion of 84 studies. The majority of studies (123 of 134) scored unclear or high risk of bias on at least one domain, mainly study design or index test. Only 11 studies scored low risk on all domains (29–39). Most papers scored low risk on applicability concerns, reference standard (histological assessment), and flow and timing. The main findings of the risk of bias assessment are depicted in Supplementary Figures 1, A and B (detailed overview, available online).

**Figure 3.**
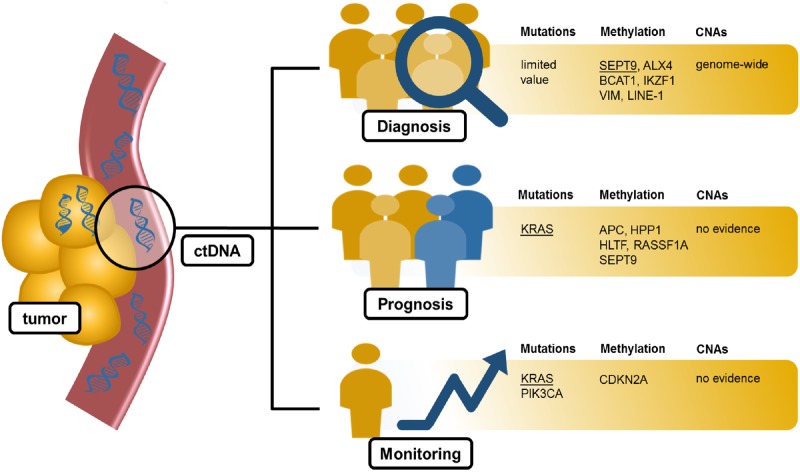
A graphical overview of the evidence for circulating tumor DNA (ctDNA) use in clinical practice. The most promising markers are presented for each clinical implication. Markers considered to be of special interest are underlined. Other markers depicted in the figure are promising but require further research. CNA = copy number alteration.

An overview of the clinical implications with the main markers of interest is provided in Figure 3.

### Accuracy of ctDNA Analysis for CRC Diagnosis

Current screening methods consist of FOBT and colonoscopy and have an overall sensitivity of 51% for individuals experiencing clinical symptoms and 19% at earlier stages (40). The present section describes ctDNA aberrations that could aid in CRC detection. Tables 1 and 2 present an overview of the identified candidate mutation and methylation markers (Table 1) and CNAs (Table 2) in ctDNA with sensitivities per stage, specificities, and concordance rates.

**Table 1.** An overview of the sensitivity and specificity for CRC detection of all ctDNA mutation, hypermethylation, and hypomethylation markers included in this review*

Marker	Sensitivity					Specificity	Concordance with primary tumor
	Stage I	Stage II	Stage III	Stage IV	Stage not reported		
Mutation							
*APC* (41–48)	0–50%	6–57%	3–46%	15–75%	14–18%	NA	16–100%
*BRAF* (34,35,42,45,48–53)	50%	0–9%	33%	3–29%	2–12%	NA	33–100%
*ERBB2* (44,54)	NA				5–9%	NA	NA
*KRAS* (32,34,35,41–46,48–53,55–64)	0–67%	3–46%	5–50%	5–73%	8–71%	70–100%	25–100%
*NRAS* (35,52)	NA				12%	NA	100%
*PIK3CA* (44,45,48,49,51)	NA			19%	0–21%	NA	0%–100%
*tp53* (41–46,48)	0–25%	22–30%	17–49%	38–67%	6–50%	100%	14–100%
Hypermethylation							
*AKAP12* (65)	NA				48%	92.0%	NA
*ALX4* (66–70)	75%	83%	82%	100%	29–83%	66–99%	NA
	30%		60%				
*APC* (67,71–74)	24%		60%	54%	20–57%	68–100%	50%
*BCAT1* (31,75)	21%	62%	68%	81%	57–65%	95–97%	NA
*BMP3* (67)	NA				29%	89%	NA
*BNC1* (67)	NA				12%	87%	NA
*BRCA1* (67)	NA				25%	78%	NA
*CDH1* (72)	NA				60%	84%	NA
*CDH4* (76)	NA				70%	100%	83%
*CDKN2A* (55,67,71,77–79)	15%	50–67%	50–67%	10–75%	9–61%	70%–96%	70–82%
*CRABP1* (75,80)	NA				50%	NA	NA
*DAPK1* (72)	50.0%	NA				74%	80%
*DLC1* (81)	36%		48%		42%	91%	NA
*ERCC1* (82)	60%			NA	NA	93%	90%
*EYA4* (80)	NA				50%	NA	NA
*FBN2* (83)	9%	7%	8%	NA	9%	NA	8%
*FGF5* (75)	NA				85%	83%	NA
*FHIT* (72,74)	NA				20–50%	84%	40%
*GATA5* (84)	46%		83%		61%	NA	NA
*GRASP* (75)	NA				54%	93%	NA
*HIC1* (67)					6%	99%	NA
*HLTF* (67,85–89)	8–20%	15–16%	9–16%	24–47%	11–30%	96–100%	41–42%
*hMLH1* (67,77,89)	27%	0–24%	25–27%	12–40%	16–29%	100%	33%
*HPP1* (85,87–90)	3–7%	0–6%	5–9%	52–53%	13–72%	NA	56%
*IKZF1* (31,75)	28%	41%	55%	94%	48–68%	95–99%	NA
*IRF4* (75)	NA				59%	96%	NA
*ITGA4* (84)	24%		54%		37%	81%	NA
*LRR3CB* (74)	NA				15%	NA	23%
*MAL* (80)					50%	NA	NA
*MGMT* (67,82)	58%			NA	6%	95–99%	94%
*MLH1* (67,77,89,91)	NA				45%	57%	33%
*NELL1* (80)				NA	33%	NA	NA
*NDRG4* (67,92)	54%		56%		9–55%	NA	NA
*NEUROG1* (67,87)	31%	28%	26%	20%	21–26%	NA	NA
*NGFR* (93)	20%	25%	36%	36%	38%	91.4%	NA
*NPTX2* (67)	NA				70%	41%	NA
*OSMR* (67,94)	74%		77%		11–75%	86–93%	79%
*p73* (77)	NA				25%	NA	NA
*PCDH10* (36)	71%		54%		63%	NA	67%
*PDX1* (75)	NA				45%	70%	NA
*PHACTR3* (67)	NA				15%	94%	NA
*PPENK* (67)	NA				10%	96%	NA
*RAR-β* (67)					25%	30%	
*RASSF1A* (67,73)	14%		47%	45%	11–34%	84–100%	NA
*RUNX3* (95)	33%		50%		42%	100%	NA
*SDC2* (67,75)	NA				24–59%	84–94%	NA
*SEPT9* (25,29,30,32,33,66,67,75, 80,93,94,96–107)	14–84%	50–100%	38–100%	68–100%	24–96%	73–97%	80–88%
	20–57%		52–70%				
	64%			NA			
*SFRP1* (67)					22%	93%	
*SFRP2* (67,84)	42%		71%		20–54%	72–82%	NA
*SHOX2* (103)	NA			44%	21%	NA	NA
*SMAD4* (72)	NA				52%	64%	NA
*SOX21* (75)	NA				80%	50%	NA
*SPG20* (67)	NA				16%	82%	NA
*SST* (67,80)	NA				30–50%	69%	NA
*TAC1* (67,80)	NA				50–53%	53%	NA
*TFPI1* (108)	NA				7%	98%	NA
*TFPI2* (67)	0%	10%	13%	58%	18%	100%	NA
*THBD* (67)	NA				10%	99%	NA
*TMEFF2* (66,93)	5%	22%	47%	45%	30–71%	90–95%	NA
*VIM* (67,109,110)	50–52%	55–67%	40%	86%	18–71%	60–93%	78%
*WIF1* (67)	NA				10%	96%	NA
*WNT5A* (67)	NA				6%	95%	NA
Hypomethylation							
*CBS* (111)	NA				56%	NA	NA
*LINE-1* (112)	63%		68%		66%	90%	NA
Panels							
Hypermethylation: *ALX4* + *BMP3* + *NPTX2* + *RARB* + *SDC2* + *SEPT9* + *VIM* + female sex + age>66 (67)	89%		NA		91%	73%	NA
Mutations: sequencing panel including *TP53* + *APC* + *KRAS* (45)	NA				100%	NA	NA
Mutations: *APC* + *KRAS* + *TP53* (46)	0%	22%	49%	67%	35%	100%	46%
Hypermethylation: *APC* + *MGMT* + *RASSF2A* + Wif-1 (86)	87%		NA			92%	NA
Hypermethylation: *BCAT1* + *IKZF1* (38)	41%	76%	59%	71%	62%	92%	NA
Hypermethylation: *ALX4* + *SEPT 9* + *TMEFF 2* (66)	NA				84%	88%	NA

* The number of studies reporting a specific marker is represented next to the target gene. If possible, the sensitivity was presented separately for each disease stage. Concordance was defined as the percentage of agreement between ctDNA analysis and mutation or methylation analysis in the primary tumor. CRC = colorectal cancer; ctDNA = circulating tumor DNA; NA = not available, for when no data were available in a specific category.

**Table 2.** An overview of the sensitivity and specificity for CRC detection of all analyzed potential ctDNA markers

				Sensitivity					
				Stage I	Stage II	Stage III	Stage IV	Overall	Specificity
Detection of any CNA (37,39,113)				41%		73%		56%	66–87%
Chr	Arm	Locus	Gene	50–100%	45–100%	45–91%	58–100%	49–96%	
Copy number gains									
1	q			20%	33%	9%	0%	17%	100%
1	p			20%	17%	0%		9%	100%
2	q			20%	17%	9%	0%	13–19%	100%
2	p			20%	33%	9%	0%	16–17%	100%
3	q			0%			0%	9%	100%
4	q			40%	17%	0%		4%	100%
5	q			0%	17%	0%		4–19%	100%
5	p			20%	17%	18%	0%	17–18%	100%
6	p	21.1	*CCND3*	0%			15%	4%	NA
6	q			0%		9%	0%	4%	100%
6	p			20%	50%	18%	0%	26%	100%
7	q	21.2	*CDK6*	0%		5%	10%	4%	NA
7	q	34	*BRAF*	0%		5%	15%	4%	NA
7	q			0%		9%	0%	4%	100%
7	p			0%	33%	9%	0%	9%	100%
8	p	11.21	*KAT6A*	NA				20%	NA
8	q	23.1	*RSPO2*	0%	0%	5%	40%	11%	NA
8	q	24.21	*MYC*	0%			35%	9%	NA
8	p	11.21	*IKBKB*	0%			20%	4%	NA
8	q			0%		18%	0%	9%	100%
9	q			0%		9%	0%	4%	100%
9	p			NA				28%	NA
10	q			0%	33%	0%		13%	100%
10	p			0%	33%	36%	0%	13–30%	100%
11	q	13.3	*CCND1*	0%			20%	4%	NA
12	p	13.33	*KDM5A*	0%			15%	4%	NA
12	p	12.1	*KRAS*	0%			15%	4%	NA
12	p			0%	33%	9%	100%	22%	100%
13	q	12.13	*CDK8*	0%			30%	8%	NA
13	q	13.1	*BRCA2*	0%			30%	8%	NA
13	q	34	*IRS2*	0%		5%	25%	8%	NA
13				0%		27%	100%	22%	100%
15				20%	17%	9%	0%	13%	100%
17				0%	33%	45%	0%	30%	100%
17	p			NA				13%	NA
18				20%	0%			4%	100%
19				0%	33%	55%	100%	39%	100%
19	q			NA				28%	NA
19	p			NA				16%	NA
20	q	13.2	*AURKA*	0%		5%	20%	13%	NA
20	q	11.23	*SRC*	0%		5%	45%	13%	NA
20				20%	0%	18%	0%	13%	100%
20	p			NA				16%	NA
21				0%	17%	0%		4%	100%
22				20%	17%	18%	0%	17%	100%
Copy number losses									
1	p			0%		9%	0%	4–16%	100%
2	p			0%		9%	0%	4%	100%
3	q			0%		9%	0%	4%	100%
3	p			0%		18%	0%	9–13%	100%
4	q			0%		9%	0%	4%	100%
4	p			20%	33%	18%	0%	22%	100%
5	q			0%		9%	0%	4%	100%
5	p			20%	33%	18%	0%	22%	100%
6	p			NA	17%	0%		4–16%	100%
6	q			NA				28%	NA
7	q			20%	0%			4–13%	100%
7	p			0%		9%	0%	4%	100%
8	q			0%	17%	0%	0%	4%	100%
8	p			20%	50%	45%	100%	25–43%	100%
9	q			0%	33%	18%	100%	22%	100%
9	p			20%	50%	27%	0%	30%	100%
10	q			0%	33%	0%		9%	100%
10	p			0%	17%	0%		4%	100%
11	q			0%	17%	9%	0%	9%	100%
11	p			0%	33%	18%	0%	17%	100%
12	p			NA				13%	NA
12	q			20%	0%	0%	0%	4–13%	100%
12	p			20%	33%	0%	0%	13%	100%
14				0%	17%	0%	0%	4%	100%
14	q			NA				25%	NA
14	p			NA				13%	NA
15				20%	17%	0%		9%	100%
16				20%	83%	9%	0%	13–26%	100%
17	p	13.1	AURKB	0%			20%	4%	NA
17	p	13.1	TP53	0%		5%	25%	8%	NA
17				20%	17%	9%	0%	17%	100%
18	q	22.2	SOCS6	0%			30%	8%	NA
18				0%	33%	55%	0%	39%	100%
19				80%	66%	9%	100%	39%	100%
20				0%	33%	9%	0%	13%	100%
21				0%		18%	0%	9%	100%
22				40%	17%	36%	0%	30%	100%

The number of studies reporting on a specific marker is represented next to the target gene. If possible, the sensitivity was presented separately for each disease stage. CNA = copy number alteration; CRC = colorectal cancer; NA = not available, no data were available in a specific category.

In general, the analysis of ctDNA mutations showed a limited sensitivity of up to 57% in stage I–III disease, although a higher sensitivity of 75% was found in stage IV CRC using analysis of *APC* mutations. Detection of CRC by use of ctDNA copy number analysis showed promising sensitivities up to 96% but was described by only three studies. Analysis of *SEPT9* hypermethylation resulted in high sensitivities (up to 100%) and specificities up to 97%. The methylation markers adenomatous polyposis coli (*APC*), vimentin (*VIM*), branched chain amino acid transaminase 1 (*BCAT1*), Aristaless-like homeobox 4 (*ALX4*), IKAROS family zinc finger 1 (*IKZF1*), and *LINE*-*1* showed potential but were described by a limited number of studies (n < 5).

#### 

##### Mutation Marker Candidates

The mutational landscape of CRC is very heterogeneous, but several well-studied hot-spot mutations in genes with a crucial role in the progression of adenoma to carcinoma are known (6). Inactivating mutations in the tumor-suppressor gene *APC* are present in 30–70% of sporadic CRC (114). *KRAS* and *BRAF* mutations are found in 30% and 10% of CRC, respectively (114). The presence of these mutations is both a reflection of tumor biology (qualitative information) and tumor burden (quantitative information). Detection of these mutations is therefore an attractive approach for cancer diagnosis.

##### KRAS

For diagnostic purposes, point mutations of the *KRAS* gene were most frequently evaluated (n = 25 articles), resulting in sensitivities between 0 and 73% for stage I–IV CRC (32,34,35,41–46,49–53,55–64,115). Fourteen studies reported a sensitivity of more than 30% using various detection methods (32,34,35,41,42,44,53,55,56,59,61,63,64,115). The largest and most recently published studies found sensitivities between 32% and 41% in patients with stage I–IV CRC using ddPCR or Intplex allele-specific PCR in plasma (34,62,64). Two recently published studies using ddPCR to analyze ctDNA from plasma (n = 150 patients) (64) and allele-specific PCR on ctDNA from serum (n = 50 patients) (62) found sensitivities of 41% and 32%, respectively, that increased to 48% and 53% in stage IV CRC. *KRAS* mutations were rarely detected in ctDNA from healthy control individuals, illustrated by specificities ranging between 70% and 100% (32,46,49,58–60,62). Technical concordance between ctDNA and solid-tumor tissue analysis was heavily influenced by the analytical platform and ranged between 25% and 100% (32,34,35,42–44,46,49,50,52,56,57,59,61–64,115). Higher concordance rates (>60%) were reported by recent studies using ddPCR in plasma (34,35,62,64). In summary, the use of *KRAS* mutation analysis in ctDNA is hampered by low sensitivities of less than 50% for detection of CRC despite relatively good specificities and concordance rates.

##### BRAF

Detection of CRC by *BRAF* mutation analysis in ctDNA was evaluated in 10 studies, all reporting relatively low sensitivities of 0–50% independently of the technique used (34,35,42,45,49–53,115). The largest cohort study on *BRAF* ctDNA analysis found a *BRAF* mutation in only one of the 115 CRC patients using nested-PCR in serum (50), and a recent study in 97 locally advanced rectal cancer patients reported *BRAF* ctDNA mutations in the plasma of only two patients using ddPCR (34). Another recent study in 21 stage IV CRC patients reported a higher sensitivity of 29% for detection of *BRAF* mutations using an NGS panel of 90 oncogenes in plasma (45). None of these studies provided data to determine specificity. Concordance rates varied heavily among studies, but the only two studies evaluating *BRAF* mutations with ddPCR found a concordance of 100% (35,53). Nevertheless, because of the low frequency of *BRAF* mutations in ctDNA of CRC patients, analysis of this aberration is not suitable for large-scale CRC screening.

##### APC

Four of eight studies investigating *APC* mutations in ctDNA reported sensitivities greater than 35% for CRC diagnosis using various detection methods (41,42,44–47,115,116). In the largest cohort (n = 133 patients), a sensitivity of 8% was found for detection of stage I–IV CRC and 15% for stage IV disease using a MassArray assay in plasma (43). A recent study showed a comparable sensitivity of 18% using an NGS panel in plasma of stage I–IV patients (44). A specificity of 100% was reported by only one study using single-strand conformation polymorphism-PCR for ctDNA detection in serum (46). The concordance for detection of *APC* mutations ranged from 16% to 100% (42–44,46,47,115). Four of the six studies describing concordance reported rates lower than 50% (43,44,46,47), none of them describing ddPCR. The low sensitivity makes *APC* an unattractive marker for CRC detection.

##### Copy Number Alterations

Aneuploidy, an abnormal number of chromosomes, is a common causal event in CRC. Several CNA patterns have been identified, including deletions of both arms of chromosome 17 and 18 in 56% and 66% of CRC patients, respectively (6). Analysis of copy numbers uses a genomewide approach so does not rely on detecting nucleotide-specific changes that may occur below the detection threshold in a cfDNA sample. Furthermore, large (>3 Mb) or high-level (≥4 copies) CNAs are absent in healthy individuals, allowing a high level of specificity (117).

So far, a limited number of studies have investigated the use of ctDNA for CRC detection. The three included studies on CNAs in blood of CRC patients are the most recent and reported inconsistent results using shallow whole-genome sequencing methods (Table 2) (39,113,118). Depending on the study, detection of CNAs was described on the level of a whole chromosome, chromosome arm, and/or a specific gene. One study reported copy number gains or losses across the whole genome in the plasma of 96% of stage I–IV CRC patients and 100% of stage IV CRC patients (113). Other studies reported lower sensitivities of 49% (39) and 56% (118) for detection in plasma of stage I–IV CRC patients. When focusing on CNAs of specific chromosomes, copy number losses on chromosome 18q and both gains and losses on chromosome 19 were found in the plasma of 39% of CRC patients (113). Furthermore, a specificity of 66–87% was reported (113,118). Because studies did not provide data to determine CNA concordance, this is not reported in Table 2. In summary, the analysis of genomewide CNAs is a promising method for noninvasive CRC detection but requires more research.

##### Methylation Marker Candidates

Hypermethylation in promotor regions of genes associated with tumorigenesis is a common phenomenon in CRC that mainly occurs in CpG islands, concentrated regions of DNA sequences susceptible to methylation. Fifteen percent of sporadic colorectal tumors are characterized by high methylation levels, referred to as CpG island methylation phenotype (119). However, CpG island methylation phenotype–negative tumors also have recurrent patterns of DNA methylation, which could allow methylation to be exploited for CRC detection (120).

##### SEPT9

Hypermethylation of the *SEPT9* promotor region was frequently investigated in large cohorts. Most of the 23 studies (25,29,30,32,33,66,67,75,80,93,94,96–107) that analyzed *SEPT9* hypermethylation by various methods demonstrated it to be among the most accurate candidate markers, reporting sensitivities greater than 50% for stage I–IV CRC (25,30,32,33,66,75,93,94,96–105,107). The analysis of *SEPT9* hypermethylation in ctDNA in plasma using quantitative methylation-specific PCR (qMSP) showed sensitivities of 61–62% in three recent large cohorts (n = 98, n = 123, and n = 187 patients) (94,104,105). Several other large-cohort studies showed potential for a commercially available test using qMSP for analysis of SEPT9 hypermethylation in plasma, reporting sensitivities between 73% and 87% for stage I–IV CRC (30,33,97,99,101,102,107). The sensitivity gradually increased with higher stages and was reported to be 100% in stage IV CRC patients in several studies (30,99,101). In most recent studies, specificities of 82–95% were found (29,67,94,99,104). The few studies describing concordance reported rates of approximately 80% (32,94,97). Overall, detection of hypermethylated *SEPT9* seems promising for CRC detection considering its high accuracy.

##### CDKN2A (p16)

All six studies evaluating cyclin-dependent kinase inhibitor 2A (CDKN2A) hypermethylation in ctDNA of CRC patients used MSP. The most recent study used qMSP and reported a sensitivity of 9% for stage I–IV CRC detection (67). Other studies published in the past decade did not find specificities exceeding 35% (71,77,78). One study reported a specificity of 96% (67). Concordance rates of 70% and 82% were described in two studies (78,79). Taken together, only a limited number of studies provided an overall picture of the potential value of *CDKN2A* hypermethylation analysis in ctDNA for CRC detection. Detection of hypermethylated *CDKN2A* by MSP does not show potential for CRC detection considering its low sensitivity.

##### HLTF

All six studies on helicase-like transcription factor (HLTF) hypermethylation analysis for the purpose of CRC detection used qMSP and described large cohorts of more than 100 patients (67,85–89). The most recent study found a sensitivity of 11% for analysis in plasma (67), which was supported by the majority of other studies describing sensitivities of less than 20% (67,85–88). Two studies reported specificities (96% and 100%) (67,89), and two studies reported concordance rates (41% and 42%) (86,88). Taken together, this candidate marker is not considered to be of value for CRC detection because of the low observed sensitivities.

##### Other Candidate Methylation Markers

Several less frequently described candidate markers presented in Table 1 showed high sensitivities, supporting their further investigation. Of particular interest for further validation are (studies with highest reported sensitivity) across stages I–IV: *ALX4* [sensitivity 83%, specificity 70% (68)], *APC* [sensitivity 57%, specificity 86% (72)], *BCAT1* [sensitivity 65%, specificity 97% (75)], *IKZF1* [sensitivity 68%, specificity 95 (76)], and *VIM* [sensitivity 71%, specificity not reported (109)]. Furthermore, hypomethylation of *LINE*-1 [sensitivity 66%, specificity 90% (112)] and cystathionine-beta-synthase (CBS) [sensitivity 56%, specificity not reported (111)] are of interest and require further study.

##### Marker Panels

The simultaneous analysis of multiple ctDNA mutation, copy number, and/or hypermethylation markers potentially results in higher accuracy for CRC detection. Most evidence arises from studies evaluating panels of hypermethylation markers. Combined analysis of *APC*, O-6-methylguanine-DNA methyltransferase (*MGMT*), Ras association domain family member 2 (*RASSF2A*), and WNT inhibitory factor 1 (*Wif-1*) hypermethylation was evaluated in 243 stage I–II CRC patients and demonstrated a sensitivity of 87% and a specificity of 92% (86). In a more recent study (n = 193 patients), a panel of the plasma hypermethylation markers *ALX4*, bone morphogenetic protein 3 (*BMP3*), neuronal pentraxin 2 (*NPTX2*), retinoic acid receptor beta (*RARB*), syndecan 2 (*SDC2*), *SEPT9*, and *VIM* analyzed with MSP showed a sensitivity of 91% for stage I–IV and 89% for stage I–II CRC using a multifactorial model accounting for sex and age (67). This study reported a specificity of 73%. The largest described panel was an NGS panel of 90 oncogenes including the most common CRC mutations. With this panel, one to six mutations were found in all 21 studied CRC patients (sensitivity 100%) without providing information on specificity (45). None of the studies reported technical concordance rates for these panels. Overall, the use of marker panels for CRC detection resulted in high accuracy.

### ctDNA for Prognostication and Treatment Selection in CRC

#### 

##### Pre therapeutic Analysis

Pre- as well as post-therapeutic ctDNA analysis have the potential to improve clinical decision making. Quantification of ctDNA before treatment could serve as a prognosticator because of a strong correlation with tumor burden. In the included studies, ctDNA analysis in therapy-naive patients allowed profiling of mutation patterns and detection of *KRAS* mutations before anti-EGFR therapy. Additionally, the presence of ctDNA was correlated with clinicopathological parameters (Figure 3), supporting its use in treatment planning. None of the included studies reported on detection of posttherapeutic ctDNA CNAs.

**Figure 4.**
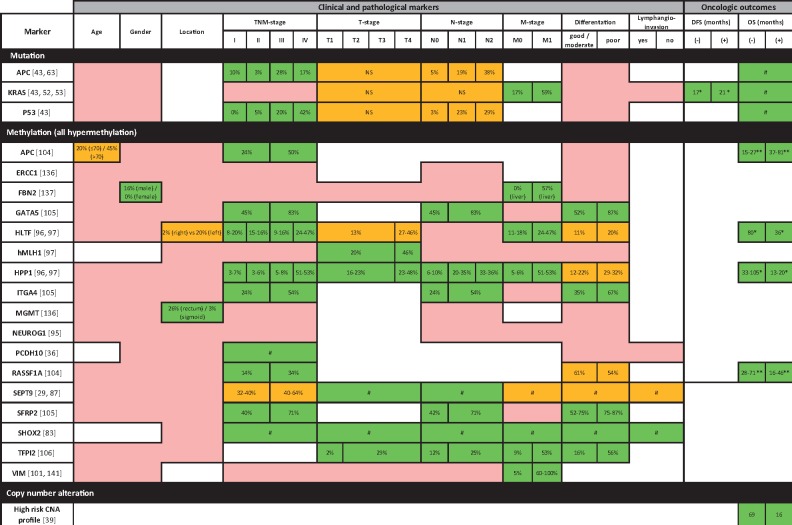
Associations between the presence of preoperative circulating tumor DNA and clinicopathological variables and oncologic outcomes. The percentage of patients with a positive marker is represented for the categories of the variables. **Green**: all studies reporting on the specific marker found statistically significant associations; **orange**: part of the studies found statistically significant and part found statistically nonsignificant (NS) associations; **pink**: all studies found statistically NS associations. The overall (OS) and disease-free survival (DFS) is presented for patients with a positive (**+**) and a negative (**−**) marker. # = no percentage of patients or median or mean OS or DFS provided, * = median, ** = mean. TNM = tumor (T), nodes (N), and metastases (M).

Quantitative analysis showed that ctDNA mutations in *KRAS*, *APC*, and *TP53* genes (41,46) and hypermethylation of multiple genes (*APC*, GATA binding protein 5 (*GATA5*), *HLTF*, hyperpigmentation, progressive, 1 (*HPP1*), integrin subunit alpha 4 (*ITGA4*), protocadherin 10 (*PCDH10*), Ras association domain family member 1 (*RASSF1A*), *SEPT9*, short stature homeobox 2 (*SHOX2*), and secreted frizzled-related protein 2 (*SFPR2*) are frequently present in patients with late-stage CRC (29,36,46,73,84,89,103,106). The detection of *KRAS* mutations (60) and hypermethylation of the *HLTF*, *HPP1*, tissue factor pathway inhibitor 2 (*TFPI2*), *SEPT9*, *SHOX2*, and *VIM* genes (89,103,108,109) in ctDNA was associated with the presence of distant metastases. Accordingly, the presence of ctDNA as detected by mutation [90-gene NGS panel (45), *KRAS*, *APC*, tumor protein P53 (*TP53*) (46,60)], copy number (113), or hypermethylation analysis [*APC*, *HLTF*, *HPP1*, *RASSF1A* (73,89,90)] was associated with worse progression-free and overall survival. Qualitative ctDNA analysis showed that presence of *KRAS* mutations in ctDNA could predict the effectiveness of targeted therapies, illustrated by an absence of clinical response to anti-EGFR therapy in stage IV CRC patients with *KRAS* mutations detected in pretherapeutic blood samples (61).

##### Post therapeutic Analysis

The detection of ctDNA after therapy could qualify patients for additional therapies by indicating residual disease or recurrence. The studies included in this review showed that the posttherapeutic detection of ctDNA mutations was correlated with poor oncologic outcome and, accordingly, may reflect (residual) tumor load after tumor resection. The detection of ctDNA using an NGS panel of 90 oncogenes after start of systemic treatment was found to be an independent risk factor for poor survival in 21 stage IV CRC patients (45). In seven CRC patients, the postoperative presence of driver gene mutations in plasma ctDNA, as detected by an 85-gene NGS panel, was associated with a poor prognosis (44). Another study (n = 60 patients) demonstrated that the persistence of serum *KRAS* mutations after surgery was associated with an increased risk of recurrence (59).

Postoperative ctDNA hypermethylation was found to be associated with poor oncologic outcome. In 79 CRC patients, *SEPT9* methylation levels dropped to barely detectable amounts after surgery in all patients except those with distant metastases or positive resection margins (103). In another study (n = 16 patients), the two patients with methylated *SEPT9* in postoperative ctDNA both presented with a recurrence during follow-up (104). Furthermore, in a study describing 82 CRC patients, postoperative detection of *SEPT9* hypermethylation in plasma was associated with increased mortality (107). Several other methylation markers were proposed as indicators of residual disease. Postoperative detection of *HPP1* hypermethylation was associated with poor survival in 337 CRC patients (90). Elevated *VIM* methylation plasma levels were associated with residual disease after surgery in patients with colorectal liver metastases, whereas CEA levels had returned to normal levels after surgery (110). Another proposed method to detect residual disease is combined analysis of plasma *BCAT1* and *IKZF1* hypermethylation. Tumor resection resulted in reduced methylation levels of these genes with complete elimination of the signal in 10 of 26 patients (31). Taken together, postoperative presence of ctDNA suggests residual disease. However, included studies consist of small cohorts and clinical validation is warranted.

### ctDNA for CRC Monitoring

Monitoring of disease by serial liquid biopsies to assess treatment response and detect recurrences during follow-up is a promising and valuable companion to current detection methods. Quantitative detection of ctDNA levels potentially allows early detection of recurrences (121). Qualitative analysis of ctDNA mutations and CNAs could find therapeutic targets and help detect therapy resistance (121).

Six studies evaluated the potential of ctDNA analysis during follow-up after surgery or during systemic treatment of CRC patients (45,55,59–61,115), all of which had small sample sizes. Five studies reported data on ctDNA mutation analysis (45,59–61,115) and one study investigated a combination of hypermethylation and mutation markers (55). No articles reported on CNAs for the use of CRC patient monitoring.

An increase in ctDNA levels, as detected by an NGS panel of 90 oncogenes, could detect resistance to chemotherapy (45). Additionally, quantitative analysis of *KRAS* mutations allowed detection of recurrences with 100% sensitivity in patients with *KRAS*-positive solid tumors (60,61,115) and improved monitoring compared with current diagnostic modalities (61). In three of seven metastatic CRC patients with a recurrence, reappearance of plasma *KRAS* mutations was detected before a diagnosis could be made using conventional methods. Moreover, in eight patients with acquired resistance during anti-EGFR therapy, *KRAS* mutations were detectable in plasma 3 months before disease progression was seen on CT scans (61). Furthermore, newly diagnosed *KRAS* and phosphatidylinositol-4,5-bisphosphate 3-kinase catalytic subunit alpha (*PIK3CA*) mutations were found up to 4 months before radiological progression in two stage IV CRC patients receiving systemic therapy (115). The combined analysis of *KRAS* mutations and *CDKN2A* methylation analysis in plasma of CRC patients increased diagnostic accuracy (55). At this moment, however, conclusions of all studies are hampered by small sample sizes.

## Discussion

Analysis of ctDNA in peripheral blood samples, so-called liquid biopsies, has the potential to realize early-stage detection of CRC and serve as a prognostic, predictive, and monitoring tool. The present systematic review is the first to evaluate the use of the most promising types of ctDNA analysis in a clinical setting. To date, the highest accuracy for CRC detection has been obtained by *SEPT9* hypermethylation analysis, especially in combined panels. For diagnostic purposes, analysis of single ctDNA mutations does not yet allow for clinical decision making. For the purposes of prognostication and disease monitoring, the most robust results were obtained by consecutive sampling and subsequent *KRAS* mutation ctDNA analysis. The analysis of CNAs could be promising for clinical use as well but is still in its infancy.

The present findings provide a starting point for implementation of ctDNA analysis into the clinic by setting out promising candidate markers. The high sensitivities of up to 100% and specificities of up to 97% of *SEPT9* methylation ctDNA analysis suggest a diagnostic role for this candidate marker. Even higher sensitivities could theoretically be obtained in combination with other promising methylation markers such as *APC*, *VIM*, *BCAT1*, *ALX4*, *IKZF1*, and *LINE*-*1*. Cancer detection through copy number analysis in ctDNA has great potential for CRC detection, with sensitivities up to 96% and specificities up to 100%. However, only a small number of included studies reported on CNAs in ctDNA, hampering solid conclusions. In contrast, analysis of single-gene ctDNA mutations showed disappointing sensitivities of less than 50% with highly variable specificities so is unlikely to increase the accuracy of current screening methods. The low sensitivities are probably due to the relatively low proportion of cfDNA fragments carrying the tumor-specific mutation, described as the variant allele frequency, or due to the absolute number of mutant DNA molecules in the sample (17,122,123).

For prognostication and disease monitoring, mutation ctDNA analysis is considered the most valuable. For prognostication, pre- and posttherapeutic analyses alike of *KRAS* and *APC* mutations provided information on tumor load (quantitative analysis) and allowed molecular profiling (qualitative analysis) to guide treatment decisions by determining the indication for (neo-)adjuvant therapies (19,20,124). Owing to correlation with oncologic outcomes, ctDNA detection after tumor resection suggests the presence of residual disease undetectable with conventional methods (17). This potentially enables accurate identification of patients for adjuvant systemic therapies. Additionally, the presence of *KRAS* mutations in ctDNA could predict treatment response to anti-EGFR therapy (61). The detection of ctDNA at higher stages could result from increased shedding of ctDNA or occult micrometastases (17). For monitoring purposes, consecutive analysis during follow-up showed high accuracy for detection of recurrences in patients with known pre-therapeutic detectable *KRAS* mutations (55,59–61,115). Additionally, *KRAS* mutation analysis in ctDNA allows repeated analysis of tumor mutations to identify acquired resistance (61) and emerging potential therapeutic targets (121). In this way, ctDNA analysis could guide tailored treatment. None of the included studies investigated CNAs for monitoring of CRC. Theoretically, serial copy number analysis could be useful as well because it does not target a specific genomic site but measures across the entire genome.

Clinical implementation of liquid biopsies for population-based screening also has high potential. Limitations of current studies are the small cohorts and poorly defined or absent healthy control individuals. Moreover, there is a lack of studies focusing on detection of precursor lesions. Before widespread implementation for screening, adequately powered validation studies comparing ctDNA with the FOBT and colonoscopy are essential. The current literature on liquid biopsies for CRC mainly consists of nonrandomized retrospective studies, with only a few markers tested in validation cohorts. A technical issue is the mutational heterogeneity observed in CRC. Accurate mutation monitoring requires expensive panel-based NGS approaches to test many genes before start of therapy and subsequent consecutive analyses of specific mutations. For this process, multiple-gene testing and highly robust assays for individual mutations are warranted, impeding widespread use. However, large-scale whole-genome mutation analysis in blood as a liquid biopsy will be feasible in the near future, enabling not only monitoring of recurrences but also evaluation of clonal evolution to adjust therapeutic approaches. Cost-effectiveness analysis and clinical validation in prospective trials are currently ongoing.

To our knowledge, this is the first systematic review assessing candidate mutation, CNA, and methylation markers in blood samples for clinical use in CRC patients. These approaches could not only complement each other but also be combined to achieve higher accuracy (125). In line with the present review, the value of methylation analysis for CRC detection is supported by a systematic review reporting hypermethylation of the *APC*, neurogenin 1 (*NEUROG1*), *RASSF1A*, *RASSF2A*, *SDC2*, *SEPT9*, tachykinin precursor 1 (*TAC1*), and thrombomodulin (*THBD*) genes in ctDNA to be detectable in early-stage CRC patients (20). However, in contrast to the low sensitivities reported for ctDNA mutation analysis, in a recent review the use of *KRAS* and *APC* mutation analysis in ctDNA was advocated for early CRC detection, with particular interest in *APC* mutations because of their presence in precursor lesions (126). Notably, this review was not performed systematically and no quality assessment was performed, impeding the authors’ conclusions.

Unfortunately, it was not possible to conduct a meta-analysis because of the variability of methods. Furthermore, the use of other liquid biopsy substrates such as circulating RNAs or circulating tumor cells was beyond the scope of this study. The focus on ctDNA was chosen because it has been investigated most extensively and is proposed as the most promising reproducible method for CRC detection with high accuracy (17–19). We included analysis of copy numbers because this is a promising, novel, and relatively simple method to detect ctDNA (15,127). Moreover, we did not include studies on other ctDNA sources currently being explored, such as urine, stool, and saliva (128,129). Similarly, other noninvasive approaches to genetic diagnosis of CRC were also omitted despite widespread clinical use. For example, the FDA-approved Cologuard (Exact Sciences) test analyzes mutations and methylation changes in DNA from stool (130). However, because stool is not a source of ctDNA, it was not covered by the scope of this study.

Translation of ctDNA into clinical daily practice is still awaited. The use of ctDNA for therapy guidance has already been suggested for locally advanced rectal cancer patients (131) and could help clinicians decide whether additional intervention is required after local excision of early-stage rectal cancer (132). A prospective comparison of current guidelines for adjuvant treatment with a novel approach based on residual ctDNA should be carried out and is currently being planned for advanced rectal cancer patients (Dynamic-Rectal study— ACTRN12617001560381). Prospective combined analysis of (epi-) genomic markers integrated with other biomarker substrates such as proteomics or metabolomics could facilitate cancer detection with higher accuracy (133). Such innovative blood tests should be designed using an “-omics” approach (134), opening up potential combinations of other candidate biomarkers. Furthermore, implementation of ctDNA analysis is promoted by novel detection methods that are being developed at a rapid pace. Techniques that are currently too expensive for routine use, such as personalized ctDNA sequencing, might become feasible within years (135). However, the use of highly sensitive and specific single-locus assays such as ddPCR, which is currently a more straightforward and cost-effective method, are still expected to be relevant (136,137), particularly for repeated measurements in patients with known tumor mutations in a tissue-guided manner (18). Finally, collaboration between academia and industrial partners is becoming increasingly important for the transition of biomarkers into the clinic, but a solid cost-effectiveness analysis is key for this purpose (138).

In conclusion, the present overview of literature proposes ctDNA analysis of methylation panels including *SEPT9* as the most valuable option for CRC detection. The use of liquid biopsies for disease monitoring seems even more promising. *KRAS* mutation analysis appears of particular interest for prognostication and monitoring of CRC patients to provide treatment guidance and tailored therapies. CNAs can be detected in the blood of CRC patients at various stages. Owing to its genomewide rather than gene-specific approach, copy number analysis could potentially be useful as a companion for early detection or monitoring. However, more research is needed. Creation of approaches combining various types of ctDNA analyses could further enhance accuracy. Prospective studies, preferably in a randomized setting in which clinical decisions depend on ctDNA results of the currently proposed candidate markers, should provide the definitive evidence to bring ctDNA analysis to clinical practice.

## Funding

This work was supported by intramural funding from the VUmc-CCA Foundation, Edli Foundation, and the Weijerhorst Foundation. This research did not receive any other specific grants from funding agencies in the public or commercial sectors.

## Notes

Affiliations of authors: Department of Surgery, Cancer Center Amsterdam (SB, NRS, JMM, GK, JBT), Department of Pathology, Cancer Center Amsterdam (JJB, NCvG, RDMS, BY) and Medical Information Specialist/Literature Researcher Medical Library (JCK), Amsterdam UMC, Vrije Universiteit Amsterdam, Vrije Universiteit, Amsterdam, the Netherlands.

## Supplementary Material

pkz042_Supplementary_Data
