# Supplementary material for: Circulating Tumor DNA Analysis: Clinical Implications for Colorectal Cancer Patients. A Systematic Review
Source: JNCI Cancer Spectr. 2019 Jun 19;3(3):pkz042. doi: 10.1093/jncics/pkz042 (PMC7050033; doi:10.1093/jncics/pkz042)
Supplement: pkz042_Supplementary_Data [file pkz042_supplementary_data.pdf]

Supplementary Table 1

Search strategy for PubMed (3 December 2018)

[Mesh] = Medical subject headings

[tiab] = words in title OR abstract

Search	Query	Items found
**#5**	#4 NOT (animals[mh] NOT humans[mh])	**2,194**
**#4**	#1 AND #2 AND #3	**2,393**
**#3**	"Blood"[Mesh] OR "blood" [Subheading] OR plasma*[tiab] OR "Hematologic Tests"[Mesh] OR hematolog*[tiab] OR haematolog*[tiab] OR blood[tiab] OR serum*[tiab] OR seral*[tiab]	**4,431,122**
**#2**	"DNA Methylation"[Mesh] OR "DNA Copy Number Variations"[Mesh] OR (("DNA"[Mesh] OR dna[tiab] OR dnas[tiab] OR kras[tiab] OR braf[tiab] OR sept9[tiab] OR "sept 9"[tiab] OR cdkn2a[tiab] OR p16[tiab] OR hltf[tiab] OR alx4[tiab] OR alex4[tiab] OR bcat1[tiab] OR ikzf1[tiab] OR vim[tiab] OR pik3ca[tiab] OR tp53[tiab] OR "tp 53"[tiab] OR apc[tiab] OR cfdna*[tiab] OR ccdna*[tiab] OR ctdna*[tiab] OR ftdna*[tiab] OR fcdna*[tiab] OR cpdna*[tiab]) AND ("Methylation"[Mesh] OR "Mutation"[Mesh] OR methylati*[tiab] OR mutation*[tiab] OR (copy number*[tiab] AND (variation*[tiab] OR variant*[tiab] OR change*[tiab])) OR abberati*[tiab] OR alterati*[tiab] OR amplificati*[tiab] OR gain*[tiab] OR loss*[tiab] OR deleti*[tiab]))	**451,201**
**#1**	"Colorectal Neoplasms"[Mesh] OR "Appendiceal Neoplasms"[Mesh] OR ((carcinoma*[tiab] OR neoplas*[tiab] OR tumour*[tiab] OR adenocar*[tiab] OR adenoid*[tiab] OR tumor[tiab] OR tumora*[tiab] OR tumorb*[tiab] OR tumorc*[tiab] OR tumord*[tiab] OR tumore*[tiab] OR tumorf*[tiab] OR tumorg*[tiab] OR tumorh*[tiab] OR tumori*[tiab] OR tumorj*[tiab] OR tumork*[tiab] OR tumorl*[tiab] OR tumorm*[tiab] OR tumorn*[tiab] OR tumoro*[tiab] OR tumorp*[tiab] OR tumorq*[tiab] OR tumorr*[tiab] OR tumors*[tiab] OR tumort*[tiab] OR tumoru*[tiab] OR tumorv*[tiab] OR tumorw*[tiab] OR tumorx*[tiab] OR tumory*[tiab] OR tumorz*[tiab] OR cancer*[tiab] OR malignan*[tiab]) AND ("Intestine, Large"[Mesh:NoExp] OR "Cecum"[Mesh] OR "Colon"[Mesh] OR "Rectum"[Mesh] OR colorectal*[tiab] OR colon*[tiab] OR rectal*[tiab] OR appendi*[tiab] OR cecum*[tiab] OR coecum*[tiab] OR caecum*[tiab] OR cecal*[tiab] OR coecal*[tiab] OR caecal*[tiab] OR sigmoid*[tiab]))	**323,289**

Search strategy for Embase.com (3 December 2018)

/exp = EMtree keyword with explosion

/de = EMtree keyword without explosion

/mj = EMtree keyword as major subject

:ti,ab = words in title or abstract

Search	Query	Items found

#5	#4 NOT ('animal cell'/de OR 'animal experiment'/de OR 'animal model'/de OR 'animal tissue'/de OR 'cancer cell culture'/de OR 'case report'/de OR 'human cell'/de OR 'in vitro study'/de OR 'nonhuman'/de)	3,221
#4	#1 AND #2 AND #3	5,462
#3	'blood'/exp OR 'blood examination'/exp OR plasma*:ab,ti,kw OR hematolog*:ab,ti,kw OR haematolog*:ab,ti,kw OR blood:ab,ti,kw OR serum*:ab,ti,kw OR seral*:ab,ti,kw	5,379,508
#2	'dna methylation'/exp OR 'dna methylation assay'/exp OR 'copy number variation'/exp OR (('dna'/exp OR dna:ti,ab,kw OR dnas:ti,ab,kw OR kras:ti,ab,kw OR braf:ti,ab,kw OR sept9:ti,ab,kw OR 'sept 9':ti,ab,kw OR cdkn2a:ti,ab,kw OR p16:ti,ab,kw OR hltf:ti,ab,kw OR alx4:ti,ab,kw OR alex4:ti,ab,kw OR bcat1:ti,ab,kw OR ikzf1:ti,ab,kw OR vim:ti,ab,kw OR pik3ca:ti,ab,kw OR tp53:ti,ab,kw OR 'tp 53':ti,ab,kw OR apc:ti,ab,kw OR cfdna*:ti,ab,kw OR ccdna*:ti,ab,kw OR ctdna*:ti,ab,kw OR ftdna*:ti,ab,kw OR fcdna*:ti,ab,kw OR cpdna*:ti,ab,kw) AND ('mutation'/exp OR 'methylation'/exp OR methylati*:ti,ab,kw OR mutation*:ti,ab,kw OR (('copy number*' NEAR/3 (variation* OR variant* OR change*)):ti,ab,kw) OR abberati*:ti,ab,kw OR alterati*:ti,ab,kw OR amplificati*:ti,ab,kw OR gain*:ti,ab,kw OR loss*:ti,ab,kw OR deleti*:ti,ab,kw))	568,751
#1	'colon tumor'/de OR 'colon cancer'/exp OR 'colorectal tumor'/exp OR 'large intestine tumor'/de OR 'appendix tumor'/exp OR 'cecum tumor'/exp OR 'large intestine cancer'/exp OR 'rectum tumor'/exp OR ((carcinoma*:ab,ti OR neoplas*:ab,ti OR tumour*:ab,ti OR adenocar*:ab,ti OR adenoid*:ab,ti OR tumor*:ab,ti OR cancer*:ab,ti OR malignan*:ab,ti) AND ('large intestine'/de OR 'cecum'/exp OR 'colon'/exp OR 'rectum'/exp OR colorectal*:ab,ti OR colon*:ab,ti OR rectal*:ab,ti OR appendi*:ab,ti OR cecum*:ab,ti OR coecum*:ab,ti OR caecum*:ab,ti OR cecal*:ab,ti OR coecal*:ab,ti OR caecal*:ab,ti OR sigmoid*:ab,ti))	486,547

Search strategy for Clarivate Analytics/Web of Science Core Collection (3 December 2018)

Indexes=SCI-EXPANDED, SSCI, A&HCI, ESCI Timespan=All years

TOPIC = words in title, abstract or author keywords

NEAR/x = words near to each other, x places apart

Search	Query	Items found
#5	#4 AND #3 AND #2 AND #1	3,063
#4	TOPIC: (plasma* OR hematolog* OR haematolog* OR blood OR serum* OR seral*)	3,582,437
#3	TOPIC: (methylati* OR mutation* OR (“copy number*” AND (variation* OR variant* OR change*)) OR abberati* OR alterati* OR amplificati* OR gain* OR loss* OR deleti*)	3,332,539
#2	TOPIC: (dna OR dnas OR kras OR braf OR sept9 OR “sept 9” OR cdkn2a OR p16 OR hltf OR alx4 OR alex4 OR bcat1 OR ikzf1 OR vim OR pik3ca OR tp53 OR “tp 53” OR apc OR cfdna* OR ccdna* OR ctdna* OR ftdna* OR fcdna* OR cpdna*)	1,381,156
#1	TOPIC: (((carcinoma* OR neoplas* OR tumour* OR adenocar* OR adenoid* OR tumor* OR cancer* OR malignan*) NEAR/3 (colorectal* OR colon* OR rectal* OR appendi* OR cecum* OR coecum* OR caecum* OR cecal* OR coecal* OR caecal* OR sigmoid*)))	289,433

Supplementary Figure 1a

Risk of bias assessment according to the QUADAS-2 for all 134 initially included studies.

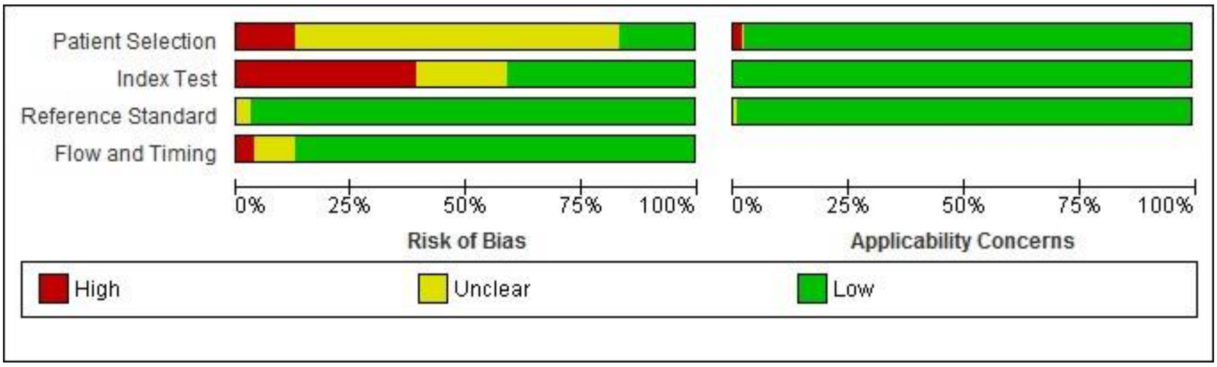

Supplementary Figure 1b

A detailed overview of the risk of bias assessment according to the QUADAS-2 for each study.

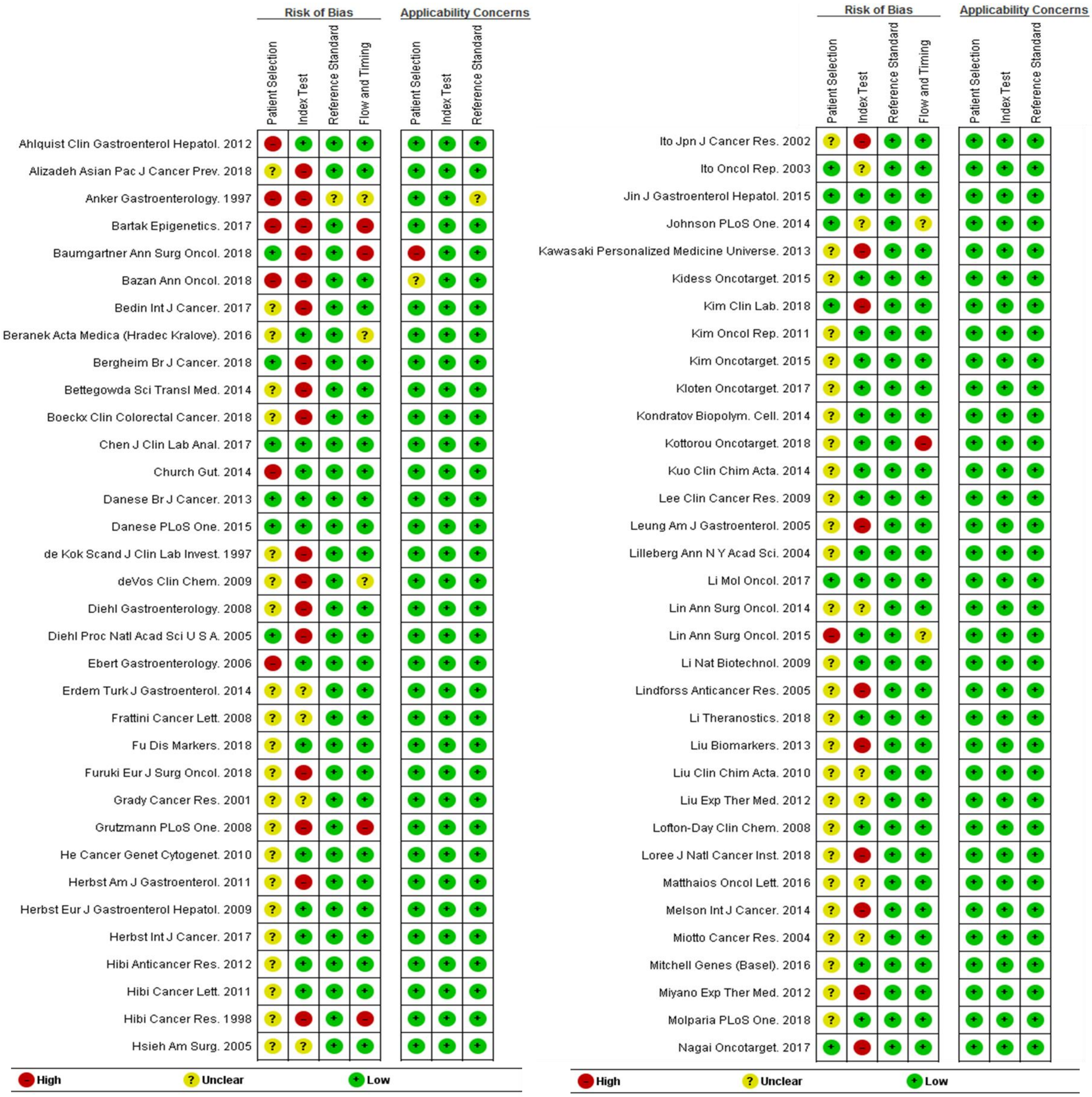

Supplementary Figure 1b (continued)

	Risk of Bias: Patient Selection	Risk of Bias: Index Test	Risk of Bias: Reference Standard	Risk of Bias: Flow and Timing	Applicability Concerns: Patient Selection	Applicability Concerns: Index Test	Applicability Concerns: Reference Standard
Nakayama Anticancer Res. 2007	?	−	+	+	+	+	+
Nakayama Anticancer Res. 2011	?	−	+	+	+	+	+
Nakayama Cancer Lett. 2002	?	−	+	+	+	+	+
Nishio Anticancer Res. 2010	?	−	+	+	+	+	+
Nunes Cancers (Basel). 2018	−	−	+	+	+	+	+
Oh J Mol Diagn. 2013	?	−	+	+	+	+	+
Olmedillas Int J Mol Sci. 2016	−	?	+	+	+	+	+
Ono Mol Oncol. 2017	?	+	+	+	+	+	+
Orntoft BMC Cancer. 2015	−	−	+	+	+	+	+
Pack Int J Colorectal Dis. 2013	−	+	+	+	+	+	+
Pedersen BMC Cancer. 2015	+	+	+	+	+	+	+
Perrone Tumori. 2014	?	?	+	+	+	+	+
Phallen Sci Transl Med. 2017	−	+	+	+	+	+	+
Philipp BMC Cancer. 2014	?	+	+	+	+	+	+
Philipp Int J Cancer. 2012	?	+	+	+	+	+	+
Potter Clin Chem. 2014	?	+	+	+	+	+	+
Pu Oncol Lett. 2013	?	?	+	+	+	+	+
Rachiglio Oncotarget. 2016	?	+	+	+	−	+	+
Rasmussen PLoS One. 2017	?	+	+	+	+	+	+
Rezvani Oncol Lett. 2017	?	−	+	+	+	+	+
Riviere Mol Cancer Ther. 2018	−	−	+	?	+	+	+
Roperch BMC Cancer. 2013	?	−	+	+	+	+	+
Ryan Gut. 2003	?	+	+	+	+	+	+
Sabbioni Mol Diagn. 2003	?	?	?	?	+	+	+
Sakai PLoS One. 2015	?	?	+	+	+	+	+
Sakamoto Cancer Epidemiol. 2010	?	?	+	+	+	+	+
Salehi Adv Biomed Res. 2015	?	?	+	+	+	+	+
Sclafani Sci Rep. 2018	+	+	+	+	+	+	+
Sefrioui Clin Biochem. 2017	+	+	+	+	+	+	+
Shalaby Gene. 2018	?	+	+	+	+	+	+
Shen Lab Med. 2010	?	−	?	?	+	+	+
Shin PLoS One. 2017	−	+	+	+	+	+	+
Shirahata Anticancer Res. 2010	?	?	+	+	+	+	+
Shirahata Anticancer Res. 2014	?	−	+	+	+	+	+

− High ? Unclear + Low

	Risk of Bias: Patient Selection	Risk of Bias: Index Test	Risk of Bias: Reference Standard	Risk of Bias: Flow and Timing	Applicability Concerns: Patient Selection	Applicability Concerns: Index Test	Applicability Concerns: Reference Standard
Song Biomark Med 2018	?	+	+	+	+	+	+
Song Epigenomics. 2017	+	−	+	+	+	+	+
Song J Cancer Res Clin Oncol. 2017	+	+	+	+	+	+	+
Sun Oncol Lett. 2018	−	−	+	+	+	+	+
Symonds Clin Epigenetics. 2018	?	−	+	+	+	+	+
Symonds Clin Transl Gastroenterol. 2016	+	+	+	+	+	+	+
Taback Ann N Y Acad Sci. 2006	?	−	+	+	+	+	+
Tanzer PLoS One. 2010	?	+	+	+	+	+	+
Thomsen Cancer Med. 2017	−	−	+	+	+	+	+
Tian Int J Biol Markers. 2017	+	−	+	+	+	+	+
Toledo Oncotarget 2017	?	−	+	+	+	+	+
Toth PLoS One. 2012	?	+	+	+	+	+	+
Toth PLoS One. 2014	?	−	+	+	+	+	+
Wallner Clin Cancer Res. 2006	?	+	+	?	+	+	+
Wang World J Surg. 2004	?	?	+	+	+	+	+
Warren BMC Med. 2011	?	+	+	+	+	+	+
Wu Chin J Cancer Res. 2011	?	?	+	+	+	+	+
Wu J Mol Diagn. 2016	+	−	+	?	+	+	+
Xiao Oncol Lett. 2015	?	?	+	?	+	+	+
Xie Front Oncol. 2018	?	+	+	+	+	+	+
Xue Oncotarget. 2017	?	?	?	?	+	+	+
Xu J Dig Dist 2018	+	+	+	+	+	+	+
Yamada Cancer Sci. 2016	?	+	+	+	+	+	+
Yamashita PLoS One. 2014	?	−	+	+	+	+	+
Yamauchi Int J Cancer. 2018	+	−	+	+	+	+	+
Yang Biosci Rep. 2018	?	−	+	+	+	+	+
Yan World J Gastroenterol. 2014	−	?	+	+	+	+	+
Yuan Transl Cancer Res 2016	?	?	+	+	+	+	+
Zeng Mol Diagn Ther. 2017	?	−	+	+	+	+	+
Zhang World J Gastroenterol. 2015	?	+	+	+	+	+	+
Zheng Hepatogastroenterology. 2011	?	?	+	+	+	+	+
Zou Clin Cancer Res. 2002	?	?	+	+	+	+	+

− High ? Unclear + Low
